# "Tinned" Meats

**Published:** 1889-01-05

**Authors:** 


### "TINNED" MEATS.

We have no wish to raise a scare, or to interfere with a legitimate industry. But public health is of much more importance than private profit, and in the public interest we call attention to a case recently reported in a medical contemporary. The case was that of a bookbinder forty-two years of age. In youth and manhood he had enjoyed good health, and had become a foreman at his calling. His good health continued until he was about forty, and then commenced a series of symptoms which made both him and his friends miserable, and which it seemed quite impossible to account for. He became languid and unequal to his work, turned against his food, and complained of obstinate constipation with continual headache. Everybody told him how pale and wretched he was looking, and all the amateur doctors of his acquaintance promptly pronounced it a case of "liver." He became so exceedingly irritable that both wife, children, and subordinates at the workshop found it very difficult to "live with him." At last came a painful attack of gout. This was followed by symptoms of colic, with more severe constipation. Then came a still more serious attack, accompanied by great pain in the abdomen, pale face, anxious expression, and cold perspiration on the forehead. What could be the matter with the man? Some of these symptoms were similar to those produced by lead-poisoning. Besides, he had the well-marked blue line along the gums, so characteristic of poisoning by lead. But the question was, how was it possible for any lead to have got into his system? He took most of his meals at home with his wife and family, and none of them were ill. If there were any lead in the water he drank or in the food he ate, why did none of the other seven or eight members of the family show any signs of harm? At last the "murder" came out. He was in the habit of eating large quantities of tinned tomatoes, which no other member of his family liked, or would eat. For three years he had taken about two tins a week. He preferred "tinned" to fresh tomatoes, and always had eaten those of the same brand. To make assurance doubly sure, the medical man in charge of the case handed a sample tin of the tomatoes to a hospital chemist and analyst for examination. He found the salts of tin present in the tomatoes to the extent of nearly one grain to the pound, and salts of lead in the proportion of more than a third of a grain to the pound. All the wretched and disabling symptoms of this man were attributed by the medical attendants who saw him to the poisonous salts of lead and tin he had swallowed with his "tinned" tomatoes. How many more people are suffering similar distress and pain from similar causes it is impossible to say. The *Lancet*, some time ago, asked why glass bottles should not be used instead of those made of tin and lead solder. Well may all people who care for their own and heir neighbour's health echo the question—"Why?"

